# Supplementary material for: Tissue-specific changes in size and shape of the ligaments and tendons of the porcine knee during post-natal growth
Source: PLoS One. 2019 Oct 23;14(10):e0219637. doi: 10.1371/journal.pone.0219637 (PMC6808441; doi:10.1371/journal.pone.0219637)
Supplement: S1 Table — (DOCX) [file pone.0219637.s001.docx]

**S1 Table. Tissue length.** Tissue length data presented as mean ± standard deviation [95% C.I.].

| Age  (months) | ACL Length  (mm) | PT Length  (mm) | MCL Length  (mm) | LCL Length  (mm) |
| --- | --- | --- | --- | --- |
| 0 | 8.7 ± 1.4  [7.2-10.1] | 14.2 ± 1.2  [13.0-15.4] | 7.7 ± 1.8  [5.9-9.6] | 7.8 ± 1.1  [6.6-9.0] |
| 1.5 | 16.7 ± 3.0  [13.6-19.8] | 25.8 ± 3.4  [21.6-30.0] | 12.5 ± 2.2  [10.2-14.8] | 12.5 ± 0.8  [11.7-13.3] |
| 3 | 23.1 ± 2.1  [20.9-25.3] | 41.3 ± 2.9  [38.3-44.3] | 20.8 ± 2.1  [18.6-23.0] | 22.2 ± 4.5  [17.5-26.9] |
| 4.5 | 29.2 ± 2.4  [26.7-31.7] | 54.9 ± 7.2  [47.4-62.4] | 33.0 ± 3.2  [29.7-36.3] | 29.4 ± 2.1  [27.2-31.5] |
| 6 | 30.5 ± 1.0  [29.5-31.5] | 58.0 ± 1.2  [56.5-59.5] | 34.2 ± 2.6  [31.5-36.9] | 34.5 ± 4.1  [30.3-38.8] |
| 18 | 34.2 ± 2.3  [32.3-37.1] | 73.8 ± 8.8  [64.5-83.0] | 38.7 ± 3.4  [35.1-42.3] | 35.6 ± 3.3  [32.2-39.0] |
