# Supplementary material for: Tissue-specific changes in size and shape of the ligaments and tendons of the porcine knee during post-natal growth
Source: PLoS One. 2019 Oct 23;14(10):e0219637. doi: 10.1371/journal.pone.0219637 (PMC6808441; doi:10.1371/journal.pone.0219637)
Supplement: S2 Table — (DOCX) [file pone.0219637.s002.docx]

**S2 Table. Tissue cross-sectional area.** Tissue cross-sectional area data presented as mean ± standard deviation [95% C.I.].

| Age  (months) | ACL CSA  (mm^2^) | PT CSA  (mm^2^) | MCL CSA (mm^2^) | LCL CSA  (mm^2^) |
| --- | --- | --- | --- | --- |
| 0 | 5.6 ± 0.7  [4.9-6.4] | 2.9 ± 1.2  [1.6-4.1] | 0.9 ± 0.3  [0.6-1.2] | 1.7 ± 0.4  [1.2-2.1] |
| 1.5 | 14.7 ± 4.7  [9.7-19.6] | 15.6 ± 6.6  [7.4-23.8] | 3.7 ± 1.2  [2.5-5.0] | 6.3 ± 1.6  [4.6-7.9] |
| 3 | 39.9 ± 4.5  [35.2-44.6] | 24.5 ± 3.5  [20.9-28.2] | 9.3 ± 1.8  [7.4-11.2] | 13.3 ± 1.9  [11.3-15.2] |
| 4.5 | 43.0 ± 7.5  [35.1-50.9] | 36.0 ± 7.3  [28.3-43.6] | 14.0 ± 2.3  [11.7-16.4] | 18.5 ± 4.3  [13.9-23.0] |
| 6 | 41.0 ± 6.4  [34.4-47.7] | 52.3 ± 18.9  [28.8-75.7] | 19.8 ± 2.9  [16.7-22.9] | 26.3 ± 3.6  [22.6-30.0] |
| 18 | 57.4 ± 8.9  [48.1-66.8] | 70.5 ± 19.5  [50.0-91.0] | 22.5 ± 3.5  [18.7-26.2] | 31.3 ± 5.1  [25.9-36.7] |
