# Supplementary material for: Tissue-specific changes in size and shape of the ligaments and tendons of the porcine knee during post-natal growth
Source: PLoS One. 2019 Oct 23;14(10):e0219637. doi: 10.1371/journal.pone.0219637 (PMC6808441; doi:10.1371/journal.pone.0219637)
Supplement: S3 Table — (DOCX) [file pone.0219637.s003.docx]

**S3 Table. Normalized tissue length.** Tissue length normalized as a percentage of the average 18-month old value presented as mean ± standard deviation [95% C.I.].

| Age  (months) | ACL Length  (%) | PT Length  (%) | MCL Length (%) | LCL Length  (%) |
| --- | --- | --- | --- | --- |
| 0 | 25.0 ± 4.1  [20.7-29.2] | 19.3 ± 1.5  [17.6-20.9] | 20.0 ± 4.6  [15.2-24.8] | 21.8 ±3.2  [18.5-25.2] |
| 1.5 | 48.1 ± 8.6  [39.2-57.1] | 35.0 ± 4.6  [29.2-40.7] | 32.3 ± 5.6  [26.5-38.2] | 35.2 ± 2.1  [32.9-37.4] |
| 3 | 66.5 ± 6.1  [60.1-72.9] | 56.0 ± 3.9  [51.9-60.1] | 53.7 ± 5.4  [48.1-59.4] | 62.2 ± 12.6  [49.0-75.4] |
| 4.5 | 84.1 ± 6.8  [76.9-91.3] | 74.5 ± 9.7  [84.7-64.3] | 85.3 ± 8.2  [76.7-93.9] | 82.4 ± 5.8  [76.3-88.5] |
| 6 | 87.8 ± 2.8  [84.8-90.8] | 78.6 ± 1.7  [76.6-80.7] | 88.5 ± 6.7  [81.5-95.5] | 97.0 ± 11.4  [85.0-109.0] |
| 18 | 100.0 ± 6.5  [93.2-106.8] | 100.0 ± 12.0  [87.4-112.6] | 100.0 ± 8.8  [90.7-109.3] | 100.0 ± 9.2  [90.4-109.6] |
