# Supplementary material for: Tissue-specific changes in size and shape of the ligaments and tendons of the porcine knee during post-natal growth
Source: PLoS One. 2019 Oct 23;14(10):e0219637. doi: 10.1371/journal.pone.0219637 (PMC6808441; doi:10.1371/journal.pone.0219637)
Supplement: S4 Table — (DOCX) [file pone.0219637.s004.docx]

**S4 Table. Normalized tissue cross-sectional area.** Tissue cross-sectional area normalized as a percentage of the average 18-month old value presented as mean ± standard deviation [95% C.I.].

| Age  (months) | ACL CSA  (%) | PT CSA  (%) | MCL CSA (%) | LCL CSA  (%) |
| --- | --- | --- | --- | --- |
| 0 | 9.8 ± 1.3  [8.5-11.1] | 4.1 ± 1.7  [2.3-5.8] | 4.0 ± 1.4  [2.5-5.4] | 5.3 ± 1.3  [3.9-6.7] |
| 1.5 | 25.6 ± 8.3  [16.9-34.2] | 22.2 ± 9.4  [10.5-33.8] | 16.6 ± 5.4  [10.9-22.3] | 19.8 ± 5.0  [14.5-25.0] |
| 3 | 69.5 ± 7.8  [61.3-77.7] | 34.8 ± 4.9  [29.6-40.0] | 41.5 ± 8.1  [33.0-50.1] | 42.0 ± 5.9  [35.8-48.2] |
| 4.5 | 74.8 ± 13.1  [61.2-88.6] | 51.0 ± 10.4  [40.1-61.9] | 62.5 ± 10.0  [52.0-73.0] | 58.5 ± 13.8  [44.0-72.9] |
| 6 | 71.5 ± 11.1  [59.9-83.1] | 74.1 ± 26.8  [40.9-107.4] | 88.2 ± 13.1  [74.5-102.0] | 83.1 ± 11.3  [71.3-95.0] |
| 18 | 100.0 ± 15.5  [83.7-116.3] | 100.0 ± 27.7  [71.0-129.0] | 100.0 ± 15.8  [83.4-116.6] | 100.0 ± 16.2  [82.1-116.1] |
