# Supplementary material for: Tissue-specific changes in size and shape of the ligaments and tendons of the porcine knee during post-natal growth
Source: PLoS One. 2019 Oct 23;14(10):e0219637. doi: 10.1371/journal.pone.0219637 (PMC6808441; doi:10.1371/journal.pone.0219637)
Supplement: Attachment — Submitted filename: Response to Reviewers.docx [file pone.0219637.s005.docx]

We thank the editor and reviewers for their thorough and positive review. The comments and suggestions have improved the quality of this manuscript. Please see the responses to specific comments below, and note that the page and line numbers indicated refer to the “Manuscript” document.

**Journal Requirements:**

**1. When submitting your revision, we need you to address these additional requirements. Please ensure that your manuscript meets PLOS ONE's style requirements, including those for file naming.**

Care has been taken to ensure that the style requirements were fulfilled.

**2. At this time, we request that you please report additional details in your Methods section regarding animal care, as per our editorial guidelines: 1) Please provide details of animal welfare (e.g., shelter, food, water, environmental enrichment) 2) please describe any steps taken to minimize animal suffering and distress, such as by administering analgesics, and 3) please include the method of sacrifice. Thank you for your attention to these requests.**

For item #2, no other procedures were performed on these animals, so there was no other need for analgesics. For items #1 and 3, additional text has been added to the methods section to address these points (Page 5, Lines 103-108) as follows:

“Swine were *housed in barns with* *food and water provided* according to the	management practices outlined in the Guide for the Care and Use of Agricultural	Animals in Teaching and Research and their use in the current	experimental	protocols were approved by the N.C.S.U. Institutional Animal Care and	Use	Committee (23). *Animals were euthanized by trained personnel in a manner	consistent with the AVMA Guidelines for the Euthanasia of Animals (24).”*

**Reviewer #1:**

**This is an interesting topic and important work for the field of pediatrics and orthopedics.**

We thank the reviewer for their kind words, and appreciate the feedback below.

**The second paragraph in the introduction reads a bit abstract. For example, the "pioneering work by D'Arcy Thompson" can be revised, as the comparisons made in the present paper are measurements of iso/allometric scaling, which was pioneered by Huxley. (e.g., citation for definitions of isometry/allometry should be: "Terminology of Relative Growth." J. S. Huxley & G. Teissier, Nature 137: 780–781 (1936)). The Thompson citation (12) also needs revising because of typos.**

We have revised the Thompson citation and adjusted the second paragraph as suggested, with the primary revisions reading as follows (Page 3, Lines 51-55):

“*Previous* work by D’Arcy Thompson (13), *Julian Huxley (14),* and many others,	have reported changes in the size and shape of biological tissues, resulting in the	establishment of many terms and methods for classifying objects during growth. *In*	*this work, we focus on the terms isometry and allometry as defined by Huxley and*	*how they can be used to describe morphological changes in tissues during* *skeletal*	*growth.* The terms isometry and allometry describe changes in which the growth of a	part, do or do not, match the growth of the whole, respectively (*13-15).”*

**How might the resolution differences between the 7.0T and 9.4T MRI scanner influence the quantitative outcomes for length/CSA? Validation comparisons of one time point (e.g., 1.5mo or younger) scanned using both 7.0T and 9.4T or discussion here would be helpful to explain potential variations.**

Unfortunately we are unable to scan any joints from 1.5 months or older using the 9.4T methods (due to bore size) and some features of the 0 month joints would not be detectable following the 7T methods (ligaments with thicknesses of <2 voxels at the 7T resolution). Although we are not able to add experimental comparisons of the two methods, we have adapted the limitations section of the discussion to better address this issue. Text additions as follows (Page 19, Lines 430-436):

*“The use of two scanning systems (7.0T MRI and 9.4T MRI) may have resulted	in some differences in the accuracy of direct comparisons between tissues	from 0-month specimens and all other groups. Unfortunately, due to the small	size of	some tissues in the 0-month limbs (dimensions less than 7.0T voxel	size) and the small bore size of the 9.4T scanner (less than the dimension of	the 1.5-month joints) we were unable to perform a direct comparison of images	taken from the tissues of interest across these scanners.”*

**What variations in animal size (weight) were found and how might this be related to iso/allometric changes in ligament size and length?**

Animal weight was not available for this cohort due to limitations at site of animal housing, although it would be an interesting metric to consider in future studies. Given the rapid growth of these animals, it is unlikely that variations in size would impact conclusions drawn between age groups.

**The rapid change in size/length that occurs from 1.5 to 3mo (e.g., ACL CSA) is interesting; do the tissue properties change during growth and could this influence the quantification of ligament size/length using MR? What methods were used to confirm that the MR images were accurately representing the growth of the soft tissues (e.g., dissection and measurement using calipers; assumptions based on morphology/imaging, etc)?**

Although changes in tissue properties may influence the intensity values associated with images at different ages, the process described in this manuscript only relies on the ability to distinguish the ligament tissue relative to surrounding tissues, which was clear at all ages. This being said, our group has previously assessed the accuracy of soft tissue shape in the juvenile porcine joint following the same 7T imaging and image processing methods described in this work by comparing to laser scan data of the same tissues. This represents small tissues at our lowest resolution. These comparisons were limited to tissues which hold their shape *ex vivo*, so this was done for the knee meniscus. We did not perform the study on ligament or tendon tissue which would deform substantially once the joint environment was disturbed. Nevertheless, we found that our MRI reconstructions resulted in similar geometries as the laser scans with root mean square error values of 0.6±0.1mm for overall surface points between methods. This is on the order of 1-2 voxels for our 7T scans. For our smallest ligament dimensions via 7T MRI in the current study were 20 and 27 voxels for CSA and length, respectively. Thus, we would expect the upper bound of the error of our measurements to be ~10% error, and for most larger specimens, we would expect the error to be much lower. Text reflecting these measurements has been added as follows (Page 7, Lines 139-145):

*“Comparison studies of the 3D reconstructions of in situ menisci calculated	from these MRI scans to a gold-standard surface reconstruction based on 3D	laser scans (FARO Edge ScanArm ES, Lake Mary, FL) indicated a root mean	square error (RMSE) of 0.57±0.1mm for points along the object surfaces and	an RMSE of 0.66±0.2mm for the centroid points of menisci. Any primary	sources of error translating from MRI-based measurements to physical tissue	morphometries should be systemic, resulting in little impact on our	comparisons between age groups.”*

**Reviewer #2**

**Summary: The purpose of this work was to characterize temporal and tissue-specific (allometric vs. isometric) changes in porcine ACL, MCL, LCL, and PT between 0-18 months of age in a cross-sectional study design. Tissues were segmented from MR images, and 3D mesh models were constructed. Tissue length and mid-substance cross-sectional area (CSA) were determined from the segmentation models. Ligament and PT growth was generally allometric with greater elongation than increases in CSA; this trend was most evident in ACL growth. The most rapid growth occurred from 1.5-4.5 months of age. The authors propose that this knowledge, when coupled with age-specific investigations of biochemical and mechanical tissue properties, have broad applicability to the fields of tissue engineering, computational modeling, regenerative medicine, and surgical reconstruction.**

**General Comments:**

**This was a concise study with particular relevance to the porcine ACL model. The manuscript and figures were clear, save for a few areas suggested under the “Specific Comments”, below.**

Thank you for these comments and your helpful suggestions below, we have updated the manuscript accordingly.

**One area of relevance of this work that could be better emphasized and discussed is the clinical relevance of high rate of ACL rupture during late adolescence. It would seem that the allometric changes were most dramatic in the ACL compared to the other extra-articular tissues examined. Could the authors speculate in the Discussion (along with any supporting evidence) why this may be, and whether they think it has any relevance to ACL injury risk? That is, dig in to the details of the statements in the paragraph spanning lines 360-370. This shift in emphasis relates most directly to the results presented, and would make the discussion on the implications of the results to other tissues, joints, and species secondary to the actual data presented.**

Thank you for bringing this up. The changes in the ACL certainly could have implications in this important clinical problem, and we have incorporated further discussion of the topic in the suggested section of the manuscript (Page 17, Lines 387-397):

*Furthermore, findings reported in this work specifically regarding allometric changes	in the morphometry of the ACL near the onset of adolescence may be	related to a	growing clinical demographic of ACL injuries. Rates of ACL tears and subsequent	reconstruction procedures have been rapidly increasing in skeletally immature	patients over recent years, with some of the most rapid growth in injury	incidence	occurring in patients between 10-13 years of age (39, 40). Unfortunately, these	patients have relatively poor outcomes from ACL	reconstruction, with much higher	secondary ACL tear incidence rates compared to adult patients undergoing the	same reconstruction treatments (41-45). Improving our understanding of the age-	specific size and shape of the ACL and how these parameters change with time may	enable clinicians to tailor graft selection and reconstruction techniques to improve	long-term outcomes in this young patient demographic.*

**Although the final data are convincing, it would be valuable to state whether all segmentations were performed by a single segmenter, and what the reproducibility of the masks (or model generation) was. That is, how good was intra- and/or inter-segmenter agreement?**

We have added the suggested information to the methods section as follows (Pages 6-7, Lines 132-136):

“*All models and measurements were generated and performed by a single author	(SC), and repeatability for this process for these ligaments and tendons has been	established to be highly repeatable, with intrauser correlation tests resulting in data	fitting with R^2^ values of 0.97-0.98 and interuser correlation tests resulting in data	fitting with R^2^ values of 0.84-0.99 across parameters.”*

**A general comment on the Results: a measure of data spread (either the standard deviation or 95% confidence intervals) should accompany the reporting of average values (e.g., average values throughout the first paragraph of their Results).**

Thank you for pointing this out, we have added standard deviations throughout the results section, and highlighted that the 95% confidence intervals are available in supplemental information (Pages 9-10, Lines 207-209):

*“Throughout the results section, data are presented as mean ± standard	deviation, and 95% confidence intervals are available in the Supplemental	Material.”*

**Review the formatting of the References – there are a few typos in the numbering.**

We have updated the references to account for these issues.

**Specific Comments:**

**Line 43: Suggest revising to “…morphometric and mechanical properties enabling force transmission and movement.” since ligaments and tendons do not generate force, per se – muscles do.**

Text has been edited as follows (Page 3, Line 43):

“...morphometric and mechanical properties enabling force *transmission* and	movement.”

**Line 50: The sentence ending with “… as well as mechanical loading.” requires a reference.**

Reference added as follows (Page 3, Line 50):

“...stimuli including biochemical and cell signaling as well as mechanical loading	*(12).”*

**Line 51: The first statement in the sentence on this line requires a reference: “Pioneering work by D’Arcy Thompson (12) and many others (ref(s)),…”**

Reference added as follows (Page 3, Line 53):

“...classifying objects during growth *(13-15).”*

**Lines 54-55: Suggest revising to “…and “allometry” describe changes in which the growth of a part do, or do not, match the growth…”**

Text has been edited as follows (Page 3, Line 56):

“...and “allometry” describe changes in which the growth of a part***,*** do or do not***,***	match the growth...”

**Specimen collection Lines 88-100: if known, please add the average mass +/- standard deviation of the animals comprising each age group.**

Unfortunately, animal weight is not available for this study, please see the response to Comment 3 from Reviewer 1.

**Line 153: I assume that the log transformations on the data were done to account for the non-linear growth characteristics, but the rationale should be stated explicitly.**

We have added explanations of the rationale to the methods section at the first mention of log transformations as follows (Page 8, Lines 179-180):

“*In order to account for non-linear tissue growth characteristics****,*** log-log plots (log10)	were created...”

**Line 154: The application of “established” here makes me think that the experimental data are being compared to data “established” in the literature, but I’m not sure that’s the intent – I think the authors are referring to the empirical slopes described in Figure 2B (?).**

This wording has been updated to exclude the term “established” for clarity (Page 8, Line 180):

“...comparing experimental data to ~~established~~ isometric slopes...”

**Related to the point above, it’s unclear where the isometric slope of 2 is derived from for the isometric slope of Length vs CSA. By definition, a slope of two is not isometric (i.e. not 1). Please provide a clearer explanation in the Methods.**

Additional text has been added clarifying that a slope of two represents isometric growth (as it is comparing the log of a mm^2 value to the log of a mm^1 value) instead of being a direct isometric slope (which would be 1). This relationship is commonly leveraged in bone growth studies to compare between parameters with different dimensions (Page 9, Lines 186-188):

*“Isometric slopes of 1 (length vs length, CSA vs CSA) or 2 (CSA vs length) were	used to account for differences in dimensions between mm and mm2 (17).”*

**Moving Lines 199-202 and 213-214 from the Results to the Statistical analysis section of the Methods would provide a clearer rationale and thorough explanation of the normalization step.**

We have moved the suggested text (shown below) to the methods section as advised (Page 7-8, Lines 156-161, 167-168):

“To compare across tissues within specific ages, values for length were normalized	to the average value at 18 months for each tissue. For example, at birth, the	average ACL length was 25% of the average ACL length at 18 months...Values for	CSA were also normalized to the average 18 month value for each tissue.”

**Line 282: Suggest revising statement to “… presented data that all three ligaments and the patellar tendon studied…” since data on the PCL were not presented, and only a single tendon was studied. Also, was there a rationale for not including the PCL in the analyses?**

We have revised the text as follows (Page 14, Lines 321-322):

“...*presented data showing that all three ligaments and the patellar tendon	studied..*.”

Regarding the PCL, we did not include this tissue in our analysis because of the significant, and sharp, change in angle in the PCL along its length (creating anterior-posterior and superior-inferior sections). Thus, we only compared tissues with a single primary axis.

**Lines 290-291: This opening sentence requires citations.**

References have been added as follows (Page 14, Line 321):

“...this work was performed to highlight differences in growth in tissues with similar	structure in a single joint *(17, 20, 24, 27, 28).”*

**Lines 389-390: Suggest removing the “…comparisons between body segments and multiple joints” from the key conclusion statement since the study did not present these data.**

We have edited the manuscript to exclude this phrase as suggested and the statement now reads as follows (Page 20, Lines 447-448):

“...increasing our understanding of this phenomenon within comparisons between	tissues	within a single organ...”
